# Chaophilic or chaotolerant fungi: a new category of extremophiles?

**DOI:** 10.3389/fmicb.2014.00708

**Published:** 2014-12-23

**Authors:** Janja Zajc, Sašo Džeroski, Dragi Kocev, Aharon Oren, Silva Sonjak, Rok Tkavc, Nina Gunde-Cimerman

**Affiliations:** ^1^Department of Biology, Biotechnical Faculty, University of LjubljanaLjubljana, Slovenia; ^2^Department of Knowledge Technologies, Jožef Stefan InstituteLjubljana, Slovenia; ^3^Centre of Excellence for Integrated Approaches in Chemistry and Biology of Proteins (CIPKeBiP)Ljubljana, Slovenia; ^4^Department of Plant and Environmental Sciences, The Institute of Life Sciences, The Hebrew University of JerusalemJerusalem, Israel; ^5^Department of Pathology, Uniformed Services University of the Health SciencesBethesda, MD, USA

**Keywords:** xerophiles, halophiles, kosmotropes, chaotropes, magnesium chloride, calcium chloride, life limit

## Abstract

It is well known that few halophilic bacteria and archaea as well as certain fungi can grow at the highest concentrations of NaCl. However, data about possible life at extremely high concentrations of various others kosmotropic (stabilizing; like NaCl, KCl, and MgSO_4_) and chaotropic (destabilizing) salts (NaBr, MgCl_2_, and CaCl_2_) are scarce for prokaryotes and almost absent for the eukaryotic domain including fungi. Fungi from diverse (extreme) environments were tested for their ability to grow at the highest concentrations of kosmotropic and chaotropic salts ever recorded to support life. The majority of fungi showed preference for relatively high concentrations of kosmotropes. However, our study revealed the outstanding tolerance of several fungi to high concentrations of MgCl_2_ (up to 2.1 M) or CaCl_2_ (up to 2.0 M) without compensating kosmotropic salts. Few species, for instance *Hortaea werneckii, Eurotium amstelodami, Eurotium chevalieri* and *Wallemia ichthyophaga*, are able to thrive in media with the highest salinities of all salts (except for CaCl_2_ in the case of *W. ichthyophaga*). The upper concentration of MgCl_2_ to support fungal life in the absence of kosmotropes (2.1 M) is much higher than previously determined to be the upper limit for microbial growth (1.26 M). No fungal representatives showed exclusive preference for only chaotropic salts (being obligate chaophiles). Nevertheless, our study expands the knowledge of possible active life by a diverse set of fungi in biologically detrimental chaotropic environments.

## Introduction

Water is essential to life, and life can only exist within a narrow range of water availability in a particular environment, expressed as water activity (a_w_). Water activity is the effective water content expressed as its mole fraction, therefore pure water has *a*_w_ = 1, all the other solutions have *a_w_* < 1. Types and amounts of solutes present in the environment lower a_w_ to various values and exert additional effects on the growth of microorganisms—causing osmotic pressure and/or have toxic effects. The lowest a_w_ known to support life is 0.61, measured for the xerophilic fungus *Xeromyces bisporus* grown on sugar-based media (Pitt and Hocking, 2009), and also for some halophilic Archaea and Bacteria (Stevenson et al., 2014). Many fungi are able to thrive at low a_w_, especially the numerous xerophilic filamentous fungi and osmophilic yeasts that grow on drying foods or on foods with high concentrations of sugars (Pitt and Hocking, 1977, 2009).

In the past, fungi were not renowned for growth at high salt concentrations. However, after the first record of fungi as active inhabitants of solar salterns were published (Gunde-Cimerman et al., 2000), the study of halotolerant and halophilic fungi expanded. Since that time numerous fungal species thriving in extremely saline environments around the globe have been described, most of them being halotolerant and extremely halotolerant, and few are obligate halophiles (reviewed in Zajc et al., 2012). The most halophilic fungus known to date is *Wallemia ichthyophaga* as it requires at least 10% NaCl and grows also in solutions saturated with NaCl (Zalar et al., 2005; Zajc et al., 2014).

Fungi have some common characteristics of osmotolerance, for instance they all employ the compatible solutes strategy: they balance the osmotic pressure of the surroundings by accumulating small organic molecules (compatible solutes), most commonly glycerol, and maintain low intracellular concentrations of salt (such as toxic Na^+^ ions) (reviewed in Gostinčar et al., 2011; Zajc et al., 2012). Sensing and responding to turgor stress (either due to organic osmolytes or due to salt) is under the control of the high osmolarity glycerol (HOG) signaling pathway in all halotolerant and halophilic fungi (Gostinčar et al., 2011). The activation of the HOG pathway results in the production of glycerol, which restores the osmotic balance of the cell (Hohmann, 2009). The cells are equipped with channels allowing for a quick expulsion of glycerol, as well as its active intake when required (Luyten et al., 1995; Ferreira et al., 2005). As the concentration of glycerol is carefully regulated, this strategy allows more flexible adaptations to changing salinity. Besides energetically costly synthesis of high concentrations of organic solutes, the cells also use much energy by using different efflux and influx systems to actively eliminate surplus ions, to preserve membrane potential, regulate intracellular pH, and maintain positive turgor of the cell. Hence, the alkali-metal cation transporters are of high importance of the osmoadaptation to extremely saline environments. In fact, the Na^+^- exporting ATPase (EnaA) is the major determinant of salt tolerance in yeasts (reviewed in Ariño et al., 2010). In addition to the above active mechanisms, fungi also employ some strategies for increasing their stress resistance that may be referred as passive—like clustering cells in compact cell clumps (Palkova and Vachova, 2006; Kralj Kunčič et al., 2010), covering the cells with abundant extracellular polysaccharides or increasing the thickness (Kralj Kunčič et al., 2010), and pigmentation/ melanization (Selbmann et al., 2005; Kogej et al., 2006) of the cell wall.

As most hypersaline environments are rich in NaCl, salt tolerance of fungi and other microorganisms, and mechanisms of adaptations were generally tested by using only NaCl as the solute. Therefore, the responses to high concentrations of other chaotropic salts remained unknown. However, other salts such as MgCl_2_ are also abundantly present in nature and can be important or even life-limiting. Salts in the environment not only lower the biologically available water and cause toxicity due to the penetration of certain cations into the cell, but they also modify structural interactions of cellular macromolecules. The Hofmeister series of ions (K^+^ > Na^+^ > Mg^2+^ > Ca^2+^; SO_4_^2−^ > HPO_4_^2−^ > Cl^−^ > NO_3_^−^ > Br^−^ > ClO_3_^−^ > I^−^ > ClO^−^_4_) describes the order of the ability of ions to salt-out or salt-in proteins (Hofmeister, 1888; Kunz et al., 2004). This phenomenon is based on direct interactions between ions and macromolecules and on interactions between ions and water molecules in the first hydration shell of the macromolecule (Zhang and Cremer, 2006). Hofmeister effects of ions on biological structures are either kosmotropic or chaotropic; chaotropes weaken electrostatic interactions and destabilize biological macromolecules, whereas the contrary is true for the kosmotropes (reviewed in Oren, 2013). The difference among the kosmotropic effect of NaCl on one hand and the chaotropic effect of MgCl_2_ and CaCl_2_ on the other hand might explain why high concentrations of Mg^2+^ and Ca^2+^ are toxic even to the most halophilic microorganisms (McGenity and Oren, 2012). However, to some extent the chaotropic effects of Mg^2+^ and Ca^2+^ can be counteracted by the presence of kosmotropic ions (Williams and Hallsworth, 2009). In fact, few halophilic Archaea can grow at high concentrations of MgCl_2_, but only in the presence of significant concentrations of NaCl (Mullakhanbhai and Larsen, 1975; Oren, 1983; Oren et al., 1995). This confirms an early study of interactions among kosmotropic and chaotropic ions on the growth of the halophilic alga *Dunaliella salina* performed by Baas Becking, who discovered that toxicity of Ca^2+^ ions was diminished in the presence of sodium ions (Baas Becking, 1934; Oren, 2011).

Two types of hypersaline brines are distinguished with respect to their origin of formation; thalassohaline and athalassohaline (Oren, 2002). Thalassohaline waters, such as marine ponds, salt marshes and solar salterns, originate by evaporation of sea water and are therefore dominated by sodium and chloride ions. During the progression of evaporation, ionic composition changes due to the consecutive precipitation of calcite (CaCO_3_), gypsum (CaSO_4_·2H_2_O), halite (NaCl), sylvite (KCl) and final carnalite (KCl·MgCl_2_·6H_2_O) after their solubilities have been surpassed (Oren, 2002, 2013). The major change in the ratio of divalent and monovalent cations occurs when the total salt concentration exceeds 300–350 g l^−1^ and most of the sodium (as halite) precipitates. In the remaining brine, so-called bittern, the dominate ion becomes Mg^2+^ (Oren, 2013).

While NaCl-rich (thalassohaline) environments are well known to support a rich biodiversity, including of fungi, very little is known about the occurrence of fungi and other microorganisms in athalassohaline, MgCl_2_- and CaCl_2_- dominated environments. Several fungi were isolated from the magnesium and calcium-rich water of the Dead Sea (Oren and Gunde-Cimerman, 2012) [~2.0 M and ~0.5 M, respectively; total dissolved salts concentration ~350 g l^−1^ (Oren, 2013); water activity ~0.683 (at 35°C) (Hallsworth, personal communication)]. However, most frequently isolation media were supplemented with different NaCl concentrations (reviewed in Oren and Gunde-Cimerman, 2012) rather than with chaotropic ions such as magnesium and calcium. Recently fungal strains were isolated from the bittern brines of solar salterns (Sonjak et al., 2010), an environment earlier considered sterile due to the high concentrations of magnesium salts (Javor, 1989). These fungal strains showed elevated tolerance to MgCl_2_, a phenomenon not yet reported for fungi. This raised the issue of the existence of chaophiles among extremophilic fungi. To address the question whether chaotolerant/chaophilic fungi may exist, we have examined a range of them both from bitterns, the Dead Sea and other extreme environments, as well as reference strains from culture collections for their ability to grow at high concentrations of various chaotropic as well as kosmotropic salts.

## Material and methods

### Fungal strains

The fungal strains studied (listed in Table 1) include culture collection strains known for their halotolerance and/or xerotolerance, and reference strains not known to be derived from hypersaline or dry environments. In addition, we tested strains isolated from bitterns of the Sečovlje (Slovenia) solar salterns. All fungal strains used are maintained in the Ex Culture Collection of the Department of Biology, Biotechnical Faculty, University of Ljubljana (Infrastructural Centre Mycosmo, MRIC UL, Slovenia).

**Table 1:** **The highest concentrations of various salts for a list of fungi**.

**Strain accession no**.	***Genus***	***Species***	**Habitat**	**The highest concentration of salt with observable growth (M)**						**Type of salt with the lowest a_w_ supporting growth**	**The lowest a_w_ supporting growth (value)**
				**NaCl**	**KCl**	**MgCl_2_**	**CaCl_2_**	**NaBr**	**MgSO_4_**		
EXF-2277	*Acremonium*	*strictum*	Salterns	2	3	0.75	0.5	2	3	KCl	0.885
EXF-174	*Alternaria*	*alternata*	Salterns	3	3	0.75	1.2	2.5	1.5	NaCl	0.884
EXF-2340	*Alternaria*	*arborescens*	Salterns	2	3	1.5	1	2	3	KCl	0.885
EXF-2332	*Alternaria*	*infectoria*	Salterns	2	2.5	1.5	1.2	2.5	3	MgSO_4_	0.886
EXF-1730	*Alternaria*	sp.	Dead Sea	2.5	3	0.75	1.2	2	2	KCl	0.885
EXF-2318	*Alternaria*	*tenuissima*	Salterns	3	3	1.5	1.2	2	3	NaCl	0.884
EXF-5007	*Aspergillus*	*caespitosus*	Salterns	4	4.5	1.9	1.5	3	3	NaCl	0.825
EXF-6616	*Aspergillus*	*candidus*	Salterns	4	4.5	1.5	1.2	3.5	2.5	NaCl	0.825
EXF-6615	*Aspergillus*	*flavipes*	Salterns	3.5	4.5	1.5	2	3	2.5	CaCl_2_	0.830
EXF-1751	*Aspergillus*	*flavus*	Dead Sea	3	4	1.6	1.5	2.5	3	KCl	0.849
EXF-1760	*Aspergillus*	*niger*	Dead Sea	3.5	4	1.5	1.5	3	3	KCl	0.849
EXF-5077	*Aspergillus*	*ochraceus*	Salterns	4	4.5	1.7	1.7	3.5	3	NaCl	0.825
EXF-138 (226)	*Aspergillus*	*penicillioides*	Salterns	3.5	3.5	1.5	1.5	2.5	2	NaCl	0.854
EXF-1946	*Aspergillus*	*proliferans*	Dead Sea	4	4.5	2.1	1.9	3	3	MgCl_2_	0.808
EXF-1752	*Aspergillus*	*sclerotiorum*	Dead Sea	4	4.5	1.9	1.7	3.5	3	NaCl	0.825
EXF-1847	*Aspergillus*	*sydowii*	Dead Sea	4	3.5	1.9	2	4	3	NaBr	0.803
EXF-5006	*Aspergillus*	*tubingiensis*	Salterns	4	4	1.9	1.7	2.5	3	NaCl	0.825
EXF-189	*Aspergillus*	*ustus*	Salterns	4	4	2	1.2	3	3	MgCl_2_	0.822
EXF-4284	*Aspergillus*	*versicolor*	Salterns	4	4.5	1.5	1.7	3.5	2.5	NaCl	0.825
EXF-4303	*Aspergillus*	*wentii*	Various	3.5	4	0.75	1.5	3	2	KCl	0.849
EXF-3400	*Aureobasidium*	*melanogenum*	Freshwater	3	3	1.5	1.2	3	2	NaBr	0.861
EXF-8429	*Aureobasidium*	*melanogenum*	Freshwater	2.5	3.5	1.5	1	2	2	KCl	0.867
EXF-3382	*Aureobasidium*	*melanogenum*	Salterns	3	3.5	1.7	1.5	3	3	NaBr	0.861
EXF-3405	*Aureobasidium*	*melanogenum*	Various	2.5	4	0.75	0.5	2	2	KCl	0.849
EXF-3398	*Aureobasidium*	*namibiae*	Various	2.5	3	0.75	1.2	2	2	KCl	0.885
EXF-150	*Aureobasidium*	*pullulans*	Salterns	3	4	0.75	1.2	2	3	KCl	0.849
EXF-2481	*Aureobasidium*	*subglaciale*	Ice	2	2	0.75	1.2	1.5	2	KCl	0.922
EXF-1830	*Bjerkandera*	sp.	Dead Sea	1.5	3	0.75	0.5	0.75	1.5	KCl	0.885
EXF-6603	*Candida*	*glabrata*	Various	2	2.5	0.75	0.5	1.5	2	KCl	0.903
EXF-1987	*Candida*	*parapsilosis*	Dead Sea	3.5	4	1.6	1.7	3	2.5	KCl	0.849
EXF-517	*Candida*	*parapsilosis*	Salterns	3	3.5	1.7	1.2	3	3	NaBr	0.861
EXF-1574	*Candida*	*parapsilosis*	Ice	3	4	1.7	1.2	3	3	KCl	0.849
EXF-253	*Chaetomium*	*globosum*	Salterns	2	2.5	0.75	1	0.75	1.5	KCl	0.903
EXF-1060	*Cladosporium*	aff. *herbarum*	Dead Sea	4	4.5	1.5	1.5	3	3	NaCl	0.825
EXF-2036	*Cladosporium*	aff. *herbarum*	Dead Sea	3	4	1.7	0.5	2.5	2.5	KCl	0.849
EXF-1930	*Cladosporium*	aff. *inversicolor*	Dead Sea	2.5	2.5	0.75	1.2	4	1.5	NaBr	0.803
EXF-2034	*Cladosporium*	aff. *sphaerospermum*	Dead Sea	4	4.5	1.5	1.7	3	3	NaCl	0.825
EXF-1728	*Cladosporium*	*cladosporioides*	Dead Sea	4	3.5	0.75	1	2.5	3	NaCl	0.825
EXF-1071	*Cladosporium*	*cladosporoides*	Dead Sea	3.5	4	1.9	1.7	3.5	3	NaBr	0.832
EXF-1824	*Cladosporium*	*cladosporoides*	Dead Sea	4	4.5	2	1.7	3	3	MgCl_2_	0.822
EXF-1081	*Cladosporium*	*halotolerans*	Dead Sea	4	4.5	1.5	1.7	3	3	NaCl	0.825
EXF-2513	*Cladosporium*	*halotolerans*	Ice	3.5	4.5	1.8	1.5	3.5	3	KCl	0.831
EXF-572	*Cladosporium*	*halotolerans*	Salterns	3	4	1.5	1.2	2.5	3	KCl	0.849
EXF-1066	*Cladosporium*	*herbarum*	Dead Sea	4	4.5	1.5	1.7	3	2.5	NaCl	0.825
EXF-1000	*Cladosporium*	*langeronii*	Various	2.5	3.5	0.75	1	3	3	NaBr	0.861
EXF-2287	*Cladosporium*	*macrocarpum*	Salterns	2.5	3.5	0.75	1.2	4	2.5	NaBr	0.803
EXF-1736	*Cladosporium*	*ramotenellum*	Dead Sea	3.5	4.5	0.75	1.7	3	2	KCl	0.831
EXF-335	*Cladosporium*	*salinae*	Salterns	3	4.5	1.8	1	2.5	3	KCl	0.831
EXF-1079	*Cladosporium*	sp.	Dead Sea	4	4.5	1.5	1.7	3	2.5	NaCl	0.825
EXF-1741	*Cladosporium*	sp.	Dead Sea	2.5	4	1.5	1.5	3	2	KCl	0.849
EXF-2012	*Cladosporium*	sp.	Dead Sea	3.5	4	1.5	1.5	3	3	KCl	0.849
EXF-2015	*Cladosporium*	sp.	Dead Sea	3.5	4.5	1.5	1.7	3	2	KCl	0.831
EXF-2016	*Cladosporium*	sp.	Dead Sea	3.5	4	1.5	1.5	3	2.5	KCl	0.849
EXF-2038	*Cladosporium*	sp.	Dead Sea	4	4	1.5	1.2	3	2.5	KCl	0.825
EXF-2040	*Cladosporium*	sp.	Dead Sea	2.5	3	0.75	0.5	2	2	KCl	0.885
EXF-1986	*Cladosporium*	sp.	Dead Sea	2.5	3	0.75	1	1.5	2	KCl	0.885
EXF-1997	*Cladosporium*	sp.	Dead Sea	3.5	4.5	1.7	1.7	3	2.5	KCl	0.831
EXF-7632	*Cladosporium*	sp.	Salterns	4	4	1.8	1.7	3	3	NaCl	0.825
EXF-7634	*Cladosporium*	sp.	Salterns	4	4	1.8	1.5	3	3	NaCl	0.825
EXF-7635	*Cladosporium*	sp.	Salterns	4	4	1.8	1.5	3	3	NaCl	0.825
EXF-2037	*Cladosporium*	*sphaerospermum*	Dead Sea	4	4.5	1.5	1.5	3	2.5	NaCl	0.825
EXF-1735	*Cladosporium*	*tenellum*	Dead Sea	3.5	3.5	0.75	0.5	2.5	2.5	NaCl	0.854
EXF-1943	*Cladosporium*	*tenuissimum*	Dead Sea	3.5	4.5	2.1	1.7	2.5	3	MgCl_2_	0.808
EXF-6893	*Cryptococcus*	*albidus*	Various	3.5	3.5	1.5	1	1.5	3	NaCl	0.854
EXF-2008	*Cryptococcus*	*albidus* var. *kuetzingii*	Dead Sea	3.5	3.5	1.5	1.2	2.5	2.5	NaCl	0.854
EXF-8012	*Cryptococcus*	*diffluens*	Freshwater	2.5	2.5	1.5	1	1.5	3	MgSO_4_	0.886
EXF-3360	*Cryptococcus*	*magnus*	Ice	1.5	2.5	1.5	1.2	1.5	3	MgSO_4_	0.886
EXF-3792	*Cryptococcus*	*victoriae*	Salterns	2.5	2.5	1.5	1	2	3	MgSO_4_	0.886
EXF-1928	*Emericella*	*purpurea*	Dead Sea	4	4.5	2.1	1.9	3.5	3	MgSO_4_	0.808
EXF-1929	*Emericella*	*purpurea*	Dead Sea	3	4	1.5	1	3	2.5	KCl	0.849
EXF-1840	*Eurotium*	*amstelodami*	Dead Sea	4	4.5	1.9	1.5	2.5	3	NaCl	0.825
EXF-5620	*Eurotium*	*chevalieri*	Various	4	4.5	2.1	1.9	4	3	NaBr	0.803
EXF-1453	*Eurotium*	*herbariorum*	Salterns	4	4.5	1.9	1.7	3.5	3	NaCl	0.825
EXF-2132	*Eurotium*	*repens*	Dead Sea	4	4	2.1	1.5	2.5	2.5	MgCl_2_	0.808
EXF-441	*Eurotium*	*rubrum*	Salterns	4	4	1.9	1.7	3.5	2.5	NaCl	0.825
EXF-5573	*Exophiala*	*dermatitidis*	Freshwater	2.5	2.5	0.75	0.5	0.75	3	MgSO_4_	0.886
EXF-2060	*Exophiala*	*oligosperma*	Ice	3	3	1.5	1.2	2.5	3	NaCl	0.884
EXF-5575	*Exophiala*	*phaeomuriformis*	Freshwater	2.5	2	0.75	1	1.5	3	MgSO_4_	0.886
EXF-4024	*Exophiala*	*xenobiotica*	Ice	1.5	1.5	1.8	1.7	1.5	3	MgSO_4_	0.886
EXF-2275	*Fusarium*	aff. *equiseti*	Salterns	2	2.5	1.5	1.5	2	3	MgSO_4_	0.886
EXF-2254	*Fusarium*	*graminearum*	Salterns	2	2.5	1.5	1.2	1.5	3	MgSO_4_	0.886
EXF-132	*Hortaea*	*werneckii*	Various	5	4.5	2.1	1.2	4	3	NaCl	0.766
EXF-2682	*Hortaea*	*werneckii*	Various	5	4.5	1.9	1.5	4	3	NaCl	0.766
EXF-6651	*Hortaea*	*werneckii*	Various	5	4.5	2.1	1.7	4	3	NaCl	0.766
EXF-225 (2000)	*Hortaea*	*werneckii*	Salterns	4	4	2	1.7	4	3	NaBr	0.803
EXF-6602	*Meyerozyma*	*guilliermondii*	Various	4	4	1.5	0.5	2.5	2	NaCl	0.825
EXF-519	*Meyerozyma*	*guilliermondii*	Salterns	3	4	1.5	1.7	2.5	2.5	KCl	0.849
EXF-2006	*Meyerozyma*	*guilliermondii*	Dead Sea	3.5	3.5	1.5	1.2	2.5	2.5	NaCl	0.854
EXF-518	*Meyerozyma*	*guilliermondii*	Salterns	3	3.5	1.7	1.2	2.5	3	KCl	0.867
EXF-224	*Paecilomyces*	*farinosus*	Various	2	3.5	0.75	0.5	1.5	1.5	KCl	0.867
EXF-4108	*Penicillium*	*antarcticum*	Ice	3.5	4.5	1.5	1.5	3.5	2.5	KCl	0.831
EXF-6614	*Penicillium*	*brevicompactum*	Salterns	4	4.5	1.5	1.5	3	2	NaCl	0.825
EXF-1774	*Penicillium*	*chrysogenum*	Dead Sea	4	4.5	1.9	1.7	3.5	3	NaCl	0.825
EXF-3655	*Penicillium*	*commune*	Ice	4	4	2.1	1.2	2.5	3	MgCl_2_	0.808
EXF-1778	*Penicillium*	*corylophylum*	Dead Sea	3.5	4	1.8	1.5	3	3	KCl	0.849
EXF-1788	*Penicillium*	*crustosum*	Dead Sea	4	4.5	1.5	1.2	3	2.5	NaCl	0.825
EXF-1781	*Penicillium*	*glabrum*	Dead Sea	4	4	1.7	1.9	3	2.5	NaCl	0.825
EXF-6613	*Penicillium*	*nordicum*	Salterns	4	4	1.5	1.2	3	2.5	NaCl	0.825
EXF-3675	*Penicillum*	*palitans*	Ice	4	3.5	1.6	1	2.5	2.5	NaCl	0.825
EXF-1822	*Penicillium*	*stecki*	Dead Sea	4	4.5	1.9	1.9	3.5	3	NaCl	0.825
EXF-3663	*Phaeococcomyces*	sp.	Ice	2	3	0.75	1.2	0.75	1.5	KCl	0.885
EXF-6160	*Phaeococcomyces*	sp.	Various	2	1.5	0.75	1	2	1.5	NaBr	0.919
EXF-206	*Phaeotheca*	*triangularis*	Salterns	3.5	4	1.9	1.9	4	2.5	NaBr	0.803
EXF-657	*Phoma*	*leveillei*	Various	4	4.5	1.5	1.2	2.5	2	NaCl	0.825
EXF-513	*Rhodosporidium*	*babjevae*	Salterns	3.5	2.5	1.5	1.2	2.5	3	NaCl	0.854
EXF-3361	*Rhodosporidium*	*iobovatum*	Ice	3	3	1.5	1.5	2	3	NaCl	0.884
EXF-6425	*Rhodotorula*	*glutinis*	Various	2	2	1.5	1	1.5	3	MgSO_4_	0.886
EXF-1450	*Rhodotorula*	*laryngis*	Dead Sea	1.5	1.5	0.75	1.5	3.5	1.5	NaBr	0.832
EXF-3871	*Rhodotorula*	*mucilaginosa*	Ice	2.5	2.5	0.75	1.2	2	3	MgSO_4_	0.886
EXF-5543	*Rhodotorula*	*mucilaginosa*	Freshwater	2.5	2.5	0.75	1.2	2	3	MgSO_4_	0.886
EXF-6896	*Rhodotorula*	*mucilaginosa*	Various	1.5	4	1.5	1.2	0.75	3	KCl	0.849
EXF-1630	*Rhodotorula*	*mucilaginosa*	Ice	2.5	3	0.75	1.2	2	3	KCl	0.885
EXF-3527	*Stachybotrys*	*atra*	Various	2	3	0.75	1	1.5	1.5	KCl	0.885
EXF-1811	*Stereum*	*gausapatum*	Dead Sea	1.5	1.5	0.75	0.5	0.75	1.5	nd	nd
EXF-1806	*Trametes*	*versicolor*	Dead Sea	1.5	1.5	0.75	1	0.75	1.5	CaCl_2_	0.952
EXF-1742	*Trichoderma*	aff. *atroviride*	Dead Sea	1.5	3.5	1.5	1.2	0.75	1.5	KCl	0.867
EXF-1444	*Trichosporon*	*mucoides*	Salterns	3	2.5	1.5	1	2	1.5	NaCl	0.884
EXF-1447	*Trichosporon*	*mucoides*	Dead Sea	4	3.5	2	1.5	3.5	3	MgCl_2_	0.822
EXF-295	*Trimmatostroma*	*salinum*	Salterns	4	4	1.7	1	3.5	3	NaCl	0.825
EXF-1835	*Ulocladium*	*tuberculatum*	Dead Sea	3.5	4	2	1.2	3	3	MgCl_2_	0.822
EXF-5753	*Wallemia*	*hederae*	Various	5	4.5	1.8	1	4	2.5	NaCl	0.766
EXF-1059	*Wallemia*	*ichthyophaga*	Various	5	4.5	1.9	1	4	3	NaCl	0.766
EXF-5676	*Wallemia*	*ichthyophaga*	Various	5	4.5	2	1.2	4	3	NaCl	0.766
EXF-6069	*Wallemia*	*ichthyophaga*	Salterns	3.5	4	1.9	0.5	2	2	MgCl_2_	0.836
EXF-6070	*Wallemia*	*ichthyophaga*	Salterns	4	4.5	1.9	0.5	3	3	NaCl	0.825
EXF-8617	*Wallemia*	*ichthyophaga*	Various	4	4.5	2	1	4	3	NaBr	0.803
EXF-6068	*Wallemia*	*ichthyophaga*	Salterns	4	4.5	1.9	0.5	2	3	NaCl	0.825
EXF-994	*Wallemia*	*ichthyophaga*	Salterns	5	4.5	2.1	0.5	4	3	NaCl	0.766
EXF-753	*Wallemia*	*muriae*	Various	4	4.5	1.9	1.2	1.5	2.5	NaCl	0.825
EXF-2361	*Wallemia*	*muriae*	Various	4	4.5	1.9	1.2	1.5	3	NaCl	0.825
EXF-8359	*Wallemia*	*muriae*	Salterns	3.5	4.5	1.9	1.2	2.5	2.5	KCl	0.831
EXF-951	*Wallemia*	*muriae*	Salterns	4	4.5	1.9	0.5	4	3	NaBr	0.803
EXF-956	*Wallemia*	*sebi*	Various	4	4.5	1.9	1.5	1.5	2.5	NaCl	0.825
EXF-2298	*Wallemia*	*sebi*	Salterns	4	4.5	1.5	1.2	2.5	2.5	NaCl	0.825
EXF-9116	*Xeromyces*	*bisporus*	Various	2.5	3.5	1.6	1	2.5	2.5	KCl	0.867

### Screening of the fungal growth in media of various salt concentration and composition

Strains were first inoculated on MEA without additional salts, except for the special strains that are obligately xerophilic (*Xeromyces bisporus* FRR525/EXF-9116) or halophilic (*Wallemia ichtyophaga* EXF-1059, −5676, −994, −6068, −8617 and *W. muriae* EXF-753, −2361, −8359, −951). For the latter two species, MEA was supplemented with 2 M NaCl, whereas for *X. bisporus* MEA was supplemented with 30% (w/v) glucose. After 7–14 days of incubation at 24°C in the dark, spore suspensions were prepared using spore suspension solution (0.05% (w/v) Tween 80, 0.05% agar, 0.9% NaCl). The optical density of the spore suspensions were measured at 600 nm and adjusted to ~0.8. Spore suspension (50 μl) was added to 2 ml of the liquid Malt Extract (ME) medium (pH 7) supplemented with various salts (NaCl, KCl, NaBr, MgSO_4_, MgCl_2_, CaCl_2_) of indicated concentrations (NaCl: 2.0, 2.5, 3.0, 4.0, 5.0 M; NaBr: 1.5, 2.0, 2.5, 3.0, 3.5, 4.0 M; KCl: 2.0, 2.5, 3.0, 4.0, 4.5 M; MgCl_2_: 1.5, 1.6, 1.7, 1.8, 1.9, 2.0, 2.1 M; MgSO_4_: 2.0, 2.5, 3.0 M; CaCl_2_: 1.0, 1.2, 1.5, 1.7, 1.9, 2.0 M), and incubated in 12 ml glass test tubes (covered with metal caps and thoroughly wrapped with parafilm) at 24°C. Inoculated media were examined for visible growth (either in a form of a submerged or surface mycelium or culture turbidity due to growth of yeast cells) after 6 weeks. Negative controls (sterile medium) for each salinity and salt type were included in the experiments. Cultures were examined by light microscopy using Olympus BX51 light microscope equipped with an Olympus DP73 digital camera.

### Data analysis using machine learning

The experiments described above resulted in a dataset with a total of 135 samples. Each of the samples refers to a single fungal strain and is described with environmental conditions (considered as independent or descriptive variables), and the fungal species encountered at each sample (considered as the dependent or the target variable). More specifically, we used the following descriptive variables: habitat (with the possible values of salterns, the Dead Sea, food, freshwater, ice, human, or animal), pigmentation (non-melanized or melanized), cell morphology (filamentous, polymorphic, yeast, or clumps), the lowest a_w_ salt with observable growth, the type of salt with the lowest a_w_ still supporting growth, and the highest concentrations of various salts still supporting growth (NaCl, KCl, MgCl_2_, CaCl_2_ NaBr, and MgSO_4_). The target variable is the fungal species, described with its taxonomic rank. Taken together, the samples included information from 94 different species from 31 different genera.

The generic data analysis task that we addressed was a task of predictive modeling, relating the environmental conditions (descriptive variables) and the fungal species (target variable). We have defined seven different scenarios for analysis. The descriptive variable(s) for each were as follows: (1) the highest concentrations of salts (NaCl, KCl, MgCl_2_, CaCl_2_ NaBr and MgSO_4_), (2) habitat and salt concentrations, (3) pigmentation, morphology and salt concentrations, (4) habitat, pigmentation, morphology and salt concentrations, (5) habitat, lowest a_w_ (type of salt), lowest a_w_ (value) and salt concentrations, (6) habitat, lowest a_w_ (type of salt and lowest a_w_ value), and (7) all descriptive variables.

To analyze the data, we used the machine learning tool CLUS available for download at http://clus.sourceforge.net. More specifically, we used predictive clustering trees (PCTs) for hierarchical classification as models. PCTs are a generalization of decision trees—a machine learning approach commonly used for classification. PCTs are tree-like structures that have internal nodes and leafs. The internal nodes contain tests on the descriptive variables, while leafs represent the predictions about the target variable. PCTs can solve the general task of structured output prediction, including the specific task of hierarchical classification.

We selected PCTs to model the data because of the specific task at hand. Namely, we used the taxonomic rank of the fungi species to create a hierarchy of classes, where different species can belong to the same genera. This clearly defines the prediction task as a task of hierarchical classification. The predictive clustering trees are able to exploit the information contained in the taxonomic rank of the species during the model construction. Furthermore, the PCTs are easily interpretable predictive models. Detailed information about predictive clustering trees for hierarchical classification has been published before (Vens et al., 2008; Kocev et al., 2013; Levatić et al., 2014).

**Figure 1:**
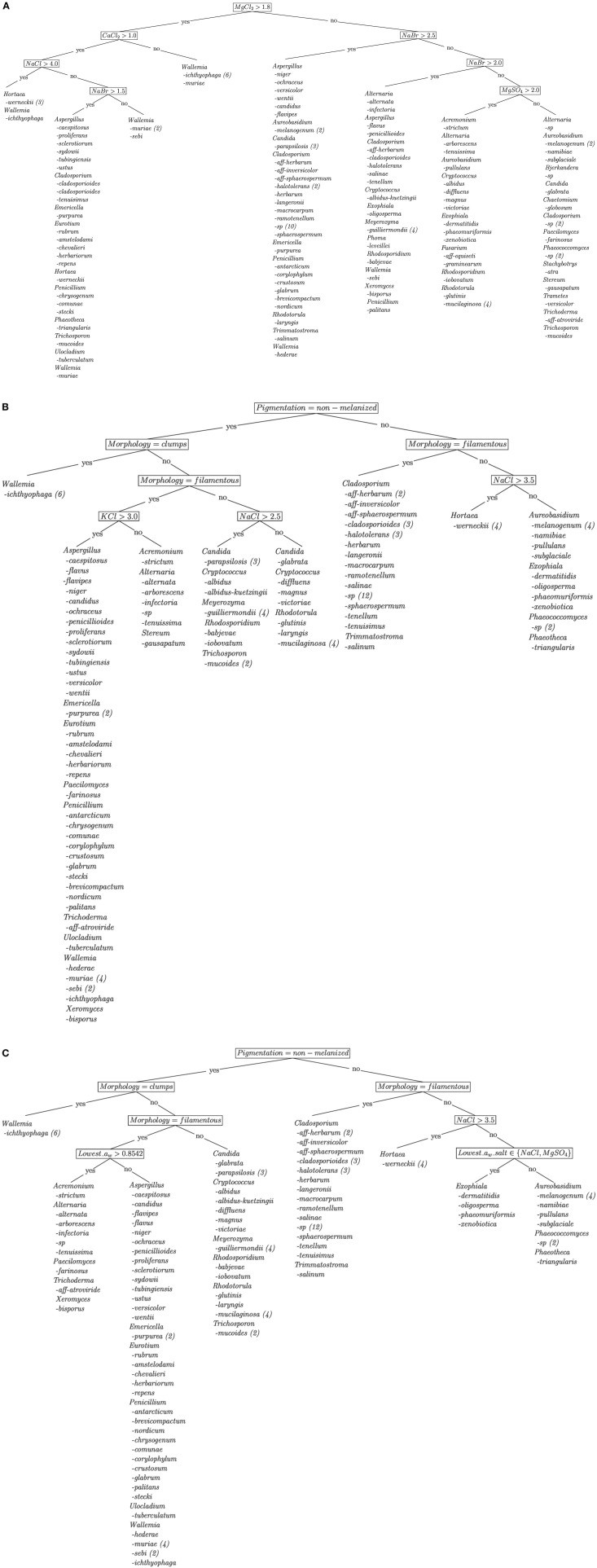
**Visualization of the decision trees of fungal species obtained by the machine learning tool CLUS**. The trees use **(A)** the highest concentrations of various salts (NaCl, KCl, MgCl_2_, CaCl_2_ NaBr and MgSO_4_); **(B)** pigmentation (melanized, non-melanized), morphology (yeast, filamentous, polymorphic, clumps) and the highest concentrations of various salts (NaCl, KCl, MgCl_2_, CaCl_2_ NaBr and MgSO_4_); and **(C)** all the descriptive variables, i.e., the highest concentrations of various salts (NaCl, KCl, MgCl_2_, CaCl_2_ NaBr and MgSO_4_), habitat (salterns, Dead Sea, freshwater, various: ice, human associated, animal associated, food), pigmentation (melanized, non-melanized), morphology (yeast, filamentous, polymorphic, clumps) and the lowest a_w_ (type of salt and value) with observable growth. The target variable was the fungal species (leaves of the decision trees). The numbers in the brackets give the numbers of strains (when more than 1) of each taxon encountered under the given conditions.

For each scenario, we have constructed a PCT for hierarchical classification. The PCTs for scenarios 1, 3, 4, and 7 are given in Figure 1. The internal nodes contain tests on individual environmental conditions (e.g., MgCl_2_ > 1.8) and leaves correspond to a specific combination of environmental conditions. In each leaf, the species encountered under the given conditions are listed.

## Results

### Screening of the fungal growth at various salts

**Figure 2:**
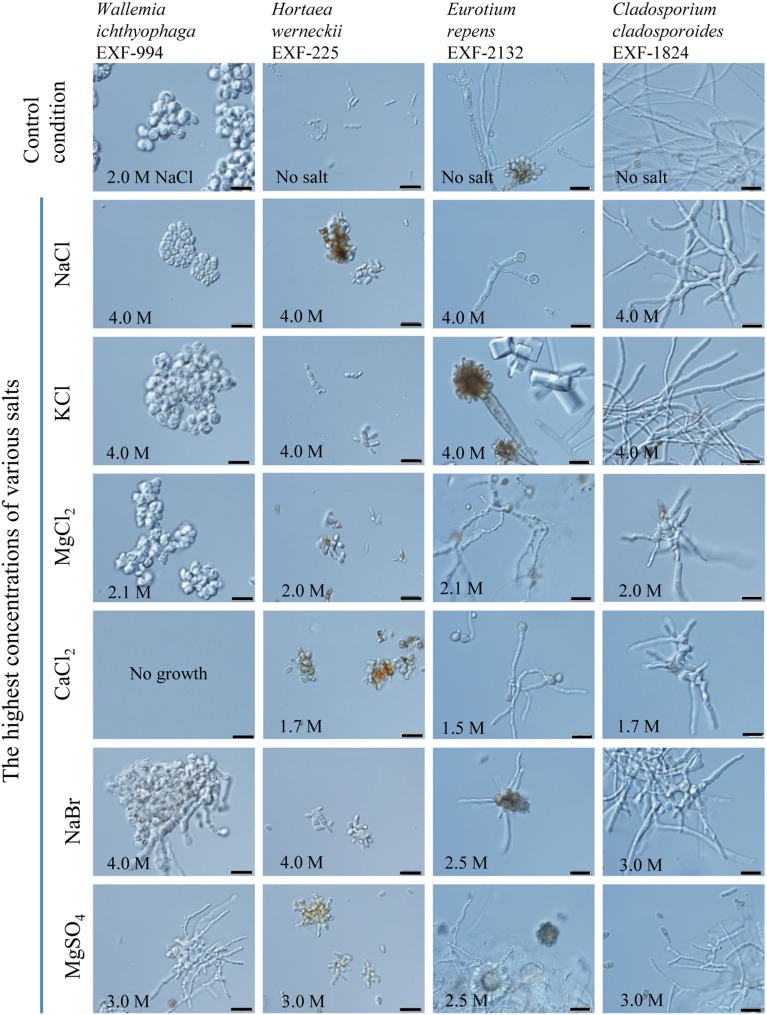
**Micromorphological characteristics of liquid culture of four strains, namely *Wallemia ichthyophaga* (EXF-994), *Hortaea werneckii* (EXF-225), *Eurotium repens* (EXF-2132) and *Cladosporium cladosporoides* (EXF-1824) grown in malt extract medium without salt (control condition) and at their highest concentrations of salt that allow growth at 24°C**. Concentrations of various salts are indicated. The scalebar represent 20 μm.

We have selected 135 fungal strains covering 94 different species and 31 genera. Amongst the genera with the highest number of strains were *Cladosporium* (23), *Aspergillus, Wallemia* (both 14) and *Penicillium* (10). The selected strains were previously isolated from different aqueous environments that contain high concentrations of salts (44 strains from salterns, 47 strains from the Dead Sea and also 13 strains from the subglacial ice) and from freshwater (6 strains). Additionally, we have included fungi from various habitats (25 representatives) including food, skin (agents of mycoses), and animals. Among the strains from the Dead Sea almost half (22) belong to the genus *Cladosporium*. We have tested growth of these strains on salts that act as kosmotropes (NaCl, KCl, and MgSO_4_) and chaotropes (CaCl_2_, MgCl_2_, and NaBr) that are present in these hypersaline environments. The highest concentrations of salts that allowed growth of individual strains are presented in the Table 1. The microscopic growth characteristics of the selected fungal representatives (cell clumps forming *W. ichthyophaga* EXF-994; a black yeast *Hortaea werneckii* EXF-225; and filamentous *Eurotium repens* EXF-2132 and *Cladosporium cladosporoides* EXF-1824) are represented in Figure 2.

### Predictive clustering trees for fungal growth

The predictive clustering trees obtained with the machine learning analysis are presented in Figure 1. When the highest concentrations of salts at which fungi were able to thrive (scenario 1) were used as the only descriptive variables, the decision tree identified chaotropic salts as the most limiting for fungal growth (Figure 1A). The most limiting turned out to be MgCl_2_ which was at the top of the decision tree, whereas CaCl_2_ and NaBr occupied internal nodes. In addition, pigmentation (melanized and non-melanized) and cell morphology (yeast, filamentous, polymorphic and clumps) (scenario 3, 4, and 7; PCT for scenarios 3 and 4 is given in Figure 1B, while for scenario 7 in Figure 1C) turned out to be key features influencing fungal distribution. Finally, when all the descriptive variables were used (including the lowest water activity and the type of salt at the lowest water activity to support growth), pigmentation was again the key variable, whereas morphology divided fungi at internal nodes and finally the lowest water activity and the type of salt in the medium with the lowest a_w_ led to the leaves (Figure 1C). Among non-melanized filamentous representatives, the ability to grow at KCl > 3.0 M and among non-melanized non-filamentous yeasts NaCl > 2.5 M turned out to be the key variables (Figure 1B). Among melanized filamentous fungi, the genus *Cladosporium* predominated, whereas for so called “black yeasts” the ability to grow at NaCl > 3.5 M was the criterion to differentiate *H. werneckii* from *Aureobasidium* sp., *Exophiala* sp., *Phaeotheca* sp. and *Phaeococcomyces* sp. (Figure 1B). When habitat was added to the other variables, almost no changes occurred in the tree (identical tree for scenarios 3 and 4, shown in Figure 1B; very similar trees for scenarios 1 and 2, shown in Figure 1A and Figure S1A).

## Discussion

Few studies have addressed the issue of the tolerance of microorganisms to chaotropic conditions over the years (reviewed in Oren, 2013). Searching for the chaophilic strains from the hypersaline deep-sea Discovery Basin, an environment with the highest salinity ever found in the marine environments—the brine is almost at saturated levels of MgCl_2_ (5.15 M) (van Der Wielen et al., 2005), did not reveal any prokaryotic representatives (Hallsworth et al., 2007). Instead, a fungus *X. bisporus*, with the lowest a_w_ limit so far reported to support life (Pitt and Hocking, 2009), was the first described species of having the preference to chaotropic conditions/solutes as it was able to grow in highly chaotropic media containing up to 7.6 M glycerol (Williams and Hallsworth, 2009) and was markedly intolerant to NaCl (Pitt and Hocking, 1977). Importantly, its growth on chaotropic solutes like MgCl_2_ and CaCl_2_ was not tested.

Indeed, fungi are promising candidates for chaophiles as they can thrive in the environments, such as crystallizer ponds of solar salterns (Gunde-Cimerman et al., 2000; Butinar et al., 2005a,b), hypersaline water of the Dead Sea (reviewed in Oren and Gunde-Cimerman, 2012) as well as the brine channels of sea ice (Gunde-Cimerman et al., 2003; Sonjak et al., 2006). As these fungi have not previously been examined for their ability to grow in media dominated by chaotropic ions, we have carried out an extensive screening of tolerance to various salts.

Our search for the chaophilic characters of fungi based on their isolation from bittern brines (Sonjak et al., 2010), residual water after the precipitation of NaCl, which is highly enriched with magnesium salts, mostly MgCl_2_. These brines were long considered sterile as high concentrations of Mg^2+^ are often toxic for biological systems. However, it was shown recently that bittern brines of the Sečovlje salterns (Slovenia) are not completely free of living microorganisms. They harbor different filamentous fungi, *Cladosporium* spp., black and other yeasts, albeit their abundance and biodiversity is low when compared to the hypersaline water of the salterns (Sonjak et al., 2010). The lower diversity and abundance might be a consequence of a combination of various factors *in situ*, such as prolonged exposure to solar radiation and magnesium, its life-limiting effect and nutrient availability.

However, the ionic composition of the bittern brine is not completely unfavorable for microbial growth despite extremely low water activity (0.737); the level of toxic ion Mg^2+^ is compensated by a relatively higher concentration of Na^+^. An outstanding discovery here was that these fungi isolated either from brine rich in MgCl_2_ or NaCl were able to grow at high concentrations of MgCl_2_(1.5 M) (Sonjak et al., 2010)—higher than previously reported for prokaryotes (1.26 M MgCl_2_) (Hallsworth et al., 2007). This observation led to the study of the ability of a list of fungi, composed of the isolates from the Dead Sea and the reference strains from our culture collection, to grow in media with low a_w_ due to high concentrations of not only kosmotropic salts (NaCl, KCl, MgSO_4_) but also chaotropic salts such as NaBr, MgCl_2_, and CaCl_2_.

Among the extremophilic fungi included in our study, 104 (almost 80% of the strains) were able to grow at concentrations of MgCl_2_ higher than 1.5 M, and among these 16 (12%) were able to grow at the highest concentrations of MgCl_2_(≥2.0 M). Next, 56 (41.5% of the strains) were capable of growth at concentrations of CaCl_2_ higher than 1.5 M, with two of these able to grow at the highest concentration (2.0 M).

The decision trees (more specifically PCTs) obtained by machine learning analysis in various scenarios (Figure 1) revealed key types of salts influencing the ability of growth of fungi. The most important salts for the limitation of fungal diversity turned out to be the chaotropic salts MgCl_2_, CaCl_2_, and NaBr, whereas KCl and NaCl appeared to be the least limiting and were not present in the nodes of the decision tree. The first decision tree (Figure 1A) revealed that 37 strains, including 11 strains of *H. werneckii* and *W. ichthyophaga* can cope with MgCl_2_ concentrations higher than 1.8 M. Here, the majority of strains of *W. ichthyophaga* were unique in their ability to tolerate the highest concentrations of MgCl_2_, but not CaCl_2_; whereas almost all strains (except for one instance) of *H. werneckii* could grow at the highest concentrations of all tested salts. Another key variable distinguishing the tested fungal strains is pigmentation, which is at the top of the decision tree using all the variables available. However, melanization is known for its role in UV and other stress responses including in osmoadaptation in halotolerant fungi (Jacobson and Ikeda, 2005; Kogej et al., 2007). Melanin impregnates the outer layer of the cell wall, this decreasing the porosity of the cell wall in order to retain more glycerol, which is most often the main compatible solute (Kogej et al., 2007). Next, cell morphology also appeared high in the decision trees. The ability to form dense clumps of meristematic cells, as observed for *W. ichthyophaga* and *Phaeotheca triangularis*, also impacts the ability of fungi to live in stressful conditions (Wollenzien et al., 1995; Palkova, 2004; Palkova and Vachova, 2006).

A simple determination of the type of salt to allow growth of individual strains at the lowest a_w_ revealed that the largest number of fungi thrived in the media with the lowest a_w_ when NaCl (52; 38.8%) or KCl (42; 31.3%) were used as the main solutes. On the contrary, less than 10% of strains were able to grow in the presence of chaotropic salts, MgCl_2_ (10; 7.5%) and CaCl_2_ (2; 1.5%), at their lowest a_w_. This again emphasizes the life-limiting effects of chaotropic salts. Whether these fungi have the preference for chaotropic salts is inconclusive, as most of them are able to grow also at the highest concentrations of other—kosmotropic—salts. Nevertheless, the fact that they are not only tolerating but growing at such high concentrations of magnesium and/or calcium salts makes these strains the most chaotolerant organisms described so far.

For comparison, the highest concentration of salts to support growth of *X. bisporus* given our results (Table 1) were 2.5 M NaCl, 3.5 M KCl, 2.5 M MgSO_4_ and 2.5 M NaBr, albeit growth was poor. In addition it was also able to grow at 1.5 M MgCl_2_ and 1 M CaCl_2_. For the inhibition of growth of *X. bisporus* the a_w_ of the medium was clearly not the determining factor—the lowest a_w_ of media tested in our study was 0.867 for medium containing 3.5 M KCl, which is far above its lowest a_w_ enabling growth in a glycerol-based medium (Pitt and Hocking, 1977). Here, it seems that high concentrations of salt, regardless of their chao- or kosmotropicity, limit the growth of *X. bisporus*, which clearly prefers sugar-based media as previously reported. Its chaophilic character on organic solutes such as glycerol (Williams and Hallsworth, 2009) must be reconsidered with caution when addressing ionic chaophilic solutes. Poor growth in the presence of salt might be a consequence of the absence of a gene coding for Na^+^-exporting ATPase (Ena) in the genome of *X. bisporus* (Leong et al., 2014), whereas this pump is present in multiple copies and/or is differentially expressed in the extremely halotolerant *H. werneckii* (Gorjan and Plemenitaš, 2006; Lenassi et al., 2013) and the halophilic *W. ichtyophaga* (Zajc et al., 2013).

*Hortaea werneckii* is a representative of the polyphyletic group of black (melanized) yeasts that have filamentous and yeast-like growth. It is able to grow across the whole range of NaCl concentrations, from 0 M to saturation, with a broad optimum from 1 M to 2.4 M NaCl (Butinar et al., 2005b), and it is thus considered to be the most extremely halotolerant fungus so far described (reviewed in Gostinčar et al., 2011). Amongst all of the melanized fungi *H. werneckii* is the most abundant in the hypersaline water of salterns (Gunde-Cimerman et al., 2000). Our screening revealed that *H. werneckii* strains are able to grow at the highest tested concentrations of salts; in media saturated with kosmotropes (5.0 M NaCl, 4.5 M KCl, 3.0 M MgSO_4_) and the highest tested concentrations of chaotropes (2.1 M MgCl_2_, 1.7 M CaCl_2_, and 4.0 M NaBr) (Table 1 and Figure 2). This exceptional ability might be linked to the redundancy of plasma membrane Na^+^ and K^+^ transporters encoded in its duplicated genome (Lenassi et al., 2013).

The genus *Aureobasidium* (de Bary) G. Arnaud is a wide-spread osmotolerant (Kogej et al., 2005) representative of black yeast associated with numerous habitats from hypersaline waters, Arctic glaciers, plant surfaces and household dust (reviewed in Gostinčar et al., 2014). In the genus, recently four new species were introduced *A. pullulans, A. melanogenum, A. subglaciale* and *A. namibiae* in Gostinčar et al. (2014). All of them are described as polyextremotolerant (Gostinčar et al., 2010, 2011) capable of surviving also hypersaline conditions (Gunde-Cimerman et al., 2000). The maximum concentrations of NaCl supporting growth of *A. pullulans* was reported to be 2.9 M NaCl (Kogej et al., 2005). Our study confirmed the upper limit of NaCl for *Aureobasidium* sp. and revealed its ability for growth at high concentrations of KCl (4.0 M) and MgSO_4_ (3.0 M), but not extremely high concentration of MgCl_2_ (lower than 1.5 M) and CaCl_2_ (up to 1.2 M). *Aureobasidium* spp. can thus be considered kosmophilic. Recent genome analysis uncovered a large repertoire of plasma-membrane transporters in the four *Aureobasidium* species (Gostinčar et al., 2014). *A. melanogenum*, which is heavily melanized, is able to grow at the highest concentrations of all salts among the four tested species of the genus *Aureobasidium*, and the least melanized *A. subglaciale* on the other hand thrives at the lowest. Here, it seems that melanization is required for the highest salt tolerance. The role of melanin in osmoadaptation was shown previously by modifying the permeability of the cell wall in order to retain the compatible solute glycerol (Jacobson and Ikeda, 2005; Kogej et al., 2006).

Representatives of the cosmopolitan genus *Cladosporium* are frequently found in habitats characterized by low a_w_, like foods preserved with sugar or salt (Samson et al., 2002), salt marshes of Egypt, in the rhizosphere of halophytic plants, and the phylloplane of Mediterranean plants (Abdel-Hafez et al., 1978). They are therefore considered xerotolerant with 0.82 being the minimal a_w_ for growth of *Cladosporium sphaerospermum* (Hocking et al., 1994). *Cladosporium* spp. are among the most abundant melanized fungi throughout the year in the solar salterns in Sečovlje (Gunde-Cimerman et al., 2000; Butinar et al., 2005b) and Cabo Rojo in Puerto Rico (Cantrell et al., 2006). Five species of the genus *Cladosporium* were isolated from the Dead Sea (reviewed in Oren and Gunde-Cimerman, 2012). The highest concentration of NaCl for *in vitro* growth of various representatives of the genus *Cladosporium* was reported to be 2.9 M to 3.5 M (Zalar et al., 2007). Strains of the genus *Cladosporium* exhibited variable tolerance to different types of salts, ranging from the lowest concentrations used in the study to the highest (Table 1). The highest growth concentrations of kosmotropic NaCl, KCl and MgSO_4_ among *Cladosporium* spp. are respectively 2.5–4.0 M, 2.5–4.5 M and 2.0–3.0 M. Two strains, *C. tenuissimum* EXF-1943 and *C. cladosporoides* EXF-1824, were able to grow at 2.0 or 2.1 M MgCl_2_ and 1.7 M CaCl_2_(Figure 2).

Species of the basidiomycetous genus *Wallemia* Johan-Olsen can be found in a wide variety of environments characterized by low a_w_ (Samson et al., 2002; Zalar et al., 2005), such as dried, salty and sweet foods, indoor and outdoor air in urban and agricultural environments, hypersaline water of the salterns on different continents and salt crystals (Zalar et al., 2005). Two species of the genus *Wallemia, W. muriae* and *W. ichthyophaga*, are obligate xerophiles with the a_w_ growth ranges 0.984–0.805 and 0.959–0.771, respectively (Zalar et al., 2005), whereas *W. sebi* is xerotolerant with the ability to grow in media without additional solutes (a_w_ growth range: 0.997–0.690) (Pitt and Hocking, 1977). However, in media supplemented with NaCl as the major solute, the lowest a_w_ for the growth of *W. sebi* was reported to be 0.80 (Zalar et al., 2005) corresponding to 4.5 M NaCl. *W. muriae* can grow up to 4.3 M NaCl, while *W. ichthyophaga* can thrive only in media with NaCl above 1.7 M, has an optimum at 2.6–3.5 M NaCl and can grow up to saturating levels of NaCl (5.2 M) (Zalar et al., 2005; Zajc et al., 2014). Here, we determined that strains of *W. ichthyophaga* grew well at highest concentrations of NaCl (above 4.0 M), NaBr (4 M) and saturated KCl and MgSO_4_, but show quite a variability when cultivated at different concentrations of chaotropes like MgCl_2_ and CaCl_2_ (Table 1 and Figure 2). A type strain from the hypersaline waters of salterns (*W. ichthyophaga* EXF-994) grew also at the 2.1 M MgCl_2_, whereas it was not able to tolerate high concentrations of calcium (not even 1 M CaCl_2_). *W. ichthyophaga* is indeed the most halophilic fungus ever described. Interestingly, its genome analysis showed that the life in extremely saline environments is possible even with low number of cation-transporter genes, and seems independent of their low transcription and non-responsiveness to variable salinity. In this case, the role of passive barriers against high salinity conditions seems crucial. The cell wall is unusually thick, the cells are joined into thick multicellular clumps and the cell-wall proteins, hydrophobins, are among the highly expressed genes in saline environments (Zajc et al., 2013).

The filamentous fungi of the order *Eurotiales*, comprised of teleomorphic genera *Eurotium* and *Emericella*, and the anamorphic *Aspergillus* and *Penicillium*, are commonly found in different salterns around the World (Cantrell et al., 2006; Butinar et al., 2011) as well as in the Dead Sea (reviewed in Oren and Gunde-Cimerman, 2012). Tolerance for high salt concentrations has been known for many food-borne species (Tresner and Hayes, 1971). The representatives of *Aspergillus* and *Penicillium* are most abundant at salinities below 1.7 M NaCl in the solar salterns (Butinar et al., 2011); however, the *in vitro* determined salinity growth ranges of the *Eurotium* spp. are broad, ranging from 0 up to 4.7 M (Butinar et al., 2005c). Given our results the highest concentrations of salts in which species from the order Eurotiales are able to thrive are highly diverse, ranging from the lowest to highest concentrations tested depending on individual strain (see Figure 2 for *Eurotium repens* EXF-2132). However, all strains were capable to grow in concentrations higher than 3.0 M NaCl, 3.5 M KCl, 2.0 M MgSO_4_, 2.5 M NaBr and even over 1.5 M MgCl_2_ and 1.2 M CaCl_2_ (except in one incident in the case of MgCl_2_ and CaCl_2_).

Few halophilic Archaea can grow at high concentrations of MgCl_2_, but only in the presence of significant concentrations of NaCl (Mullakhanbhai and Larsen, 1975; Oren, 1983; Oren et al., 1995). For instance, *Haloferax volcanii* is tolerant to high magnesium as growth is still possible at 1.4 M Mg^2+^ in the presence of 2 M Na^+^ (Mullakhanbhai and Larsen, 1975). Also, *Halobaculum gomorrense* is moderately tolerant to Mg^2+^ with optimal growth at 0.6–1.0 M Mg^2+^ in the presence of 2.1 M NaCl (Oren et al., 1995). Another archaeon isolated from the Dead Sea, *Halobacterium sodomense*, has an extremely high magnesium requirement. It grows optimally even at 1.2 M MgCl_2_ and 2.0 M NaCl and still grows, albeit poorly, at 1.8 M MgC1_2_ and 1.7 M NaCl and at 2.5 M MgC1_2_ and 0.5 M NaCl (Oren, 1983). The upper concentration of solely MgCl_2_ still supporting life was suggested to be 2.3 M and it based on the presence of specific mRNA indicators of active life, (Hallsworth et al., 2007). However, the highest concentration of MgCl_2_ (without compensating kosmotropes) showing microbial growth (after 18 months of cultivation) of deep-sea Discovery brine samples was 1.26 M (Hallsworth et al., 2007). Given the fact that it was not uncommon for fungi to thrive at concentrations of MgCl_2_ higher than 1.5 M without compensating NaCl, it is clear that fungi are truly tolerant to magnesium. Some of these were able to grow at 2.1 M MgCl_2_, a concentration that is close to the chaotropicity limit of possible life (2.3 M) (Hallsworth et al., 2007).

Fungi from diverse environments (salterns, Dead Sea, ice, freshwater and other) can not only tolerate but also thrive at high concentrations of salts, which are either kosmotropic like NaCl, KCl and MgSO_4_ or—to biological systems more toxic—chaotropic like NaBr, MgCl_2_, and CaCl_2_. A few representatives of various species, such as *H. werneckii, E. amstelodami, E. chevalieri* and *W. ichthyophaga* were able to thrive in media with the highest tested salinities of all salts (except in CaCl_2_ in case of *W. ichthyophaga*). In addition, several fungi (*Aureobasidium* spp., *Exophiala* spp.) exert a tendency toward kosmotropes, as they are able to grow at relatively high concentrations of NaCl, KCl and MgSO_4_, but not at high concentrations of chaotropes, like MgCl_2_ and CaCl_2_. However, no fungal representatives showed the preference for the highest concentrations of only chaotropic salts but not for the kosmotropic, i.e., being obligately chaophilic. Nevertheless, our study revealed many representatives of the novel group of chaophiles among fungi, which thrive well above the highest previously determined concentration of MgCl_2_. The ability to grow in the presence of high concentrations of another potent chaotrope—CaCl_2_ was addressed for the first time. This expands our knowledge of possible life performance under diverse and most extreme environmental parameters.

### Conflict of interest statement

The authors declare that the research was conducted in the absence of any commercial or financial relationships that could be construed as a potential conflict of interest.

## Supplementary material

Figure S1 **Visualization of the decision trees of fungal species obtained by machine learning tool CLUS**. The trees use **(A)** the habitat (salterns, the Dead Sea, food, freshwater, ice, human, or animal) and the highest concentrations of various salts (NaCl, KCl, MgCl_2_, CaCl_2_ NaBr and MgSO_4_); **(B)** the habitat, the lowest a_w_ (type of salt), the lowest a_w_ (value) and the highest concentrations of various salts; **(C)** the habitat, the lowest a_w_ (type of salt) and the lowest a_w_ (value). The target variable was the fungal species (leaves of the decision trees). The numbers in the brackets give the numbers of strains (when more than 1) of each taxon encountered under the given conditions.

## References

[B1] Abdel-Hafez S. Maubasher A. Abdel-Fattah H. (1978). Cellulose-decomposing fungi of salt marshes in Egypt. Folia Microbiol. (Praha). 23, 37–44. 10.1007/bf02876594 624509

[B2] Ariño J. Ramos J. Sychrova H. (2010). Alkali metal cation transport and homeostasis in yeasts. Microbiol. Mol. Biol. Rev. 74, 95–120. 10.1128/Mmbr.00042-09 20197501 PMC2832347

[B3] Baas Becking L. G. M. (1934). Geobiologie of Inleiding tot de Milieukunde. Den Haag: W.P. Van Stockum & Zoon N.V.

[B4] Butinar L. Frisvad J. C. Gunde-Cimerman N. (2011). Hypersaline waters – a potential source of foodborne toxigenic aspergilli and penicillia. FEMS Microbiol. Ecol. 77, 186–199. 10.1111/j.1574-6941.2011.01108.x 21477006

[B5] Butinar L. Santos S. Spencer-Martins I. Oren A. Gunde-Cimerman N. (2005a). Yeast diversity in hypersaline habitats. FEMS Microbiol. Lett. 244, 229–234. 10.1016/j.femsle.2005.01.043 15766773

[B6] Butinar L. Sonjak S. Zalar P. Plemenitaš A. Gunde-Cimerman N. (2005b). Melanized halophilic fungi are eukaryotic members of microbial communities in hypersaline waters of solar salterns. Botanica Marina 48, 73–79 10.1515/Bot.2005.007

[B7] Butinar L. Zalar P. Frisvad J. C. Gunde-Cimerman N. (2005c). The genus *Eurotium* - members of indigenous fungal community in hypersaline waters of salterns. FEMS Microbiol. Ecol. 51, 155–166. 10.1016/j.femsec.2004.08.002 16329864

[B8] Cantrell S. A. Casillas-Martinez L. Molina M. (2006). Characterization of fungi from hypersaline environments of solar salterns using morphological and molecular techniques. Mycol. Res. 110, 962–970. 10.1016/j.mycres.2006.06.005 16904880

[B9] Ferreira C. Van Voorst F. Martins A. Neves L. Oliveira R. Kielland-Brandt M. C.. (2005). A member of the sugar transporter family, Stl1p is the glycerol/H^+^ symporter in *Saccharomyces cerevisiae*. Mol. Biol. Cell 16, 2068–2076. 10.1091/mbc.E04-10-0884 15703210 PMC1073684

[B10] Gorjan A. Plemenitaš A. (2006). Identification and characterization of ENA ATPases HwENA1 and HwENA2 from the halophilic black yeast *Hortaea werneckii*. FEMS Microbiol. Lett. 265, 41–50. 10.1111/j.1574-6968.2006.00473.x 17034413

[B11] Gostinčar C. Grube M. de Hoog S. Zalar P. Gunde-Cimerman N. (2010). Extremotolerance in fungi: evolution on the edge. FEMS Microbiol. Ecol. 71, 2–11. 10.1111/j.1574-6941.2009.00794.x 19878320

[B12] Gostinčar C. Lenassi M. Gunde-Cimermna N. Plemenitaš A. (2011). Fungal adaptation to extremely high salt concentrations. Adv. Appl. Microbiol. 77, 71–96. 10.1016/B978-0-12-387044-5.00003-0 22050822

[B13] Gostinčar C. Ohm R. Kogej T. Sonjak S. Turk M. Zajc J.. (2014). Genome sequencing of four *Aureobasidium pullulans* varieties: biotechnological potential, stress tolerance, and description of new species. BMC Genomics 15:549. 10.1186/1471-2164-15-549 24984952 PMC4227064

[B14] Gunde-Cimerman N. Sonjak S. Zalar P. Frisvad J. C. Diderichsen B. Plemenitas A. (2003). Extremophilic fungi in arctic ice: a relationship between adaptation to low temperature and water activity. Phys. Chem. Earth 28, 1273–1278 10.1016/j.pce.2003.08.056

[B15] Gunde-Cimerman N. Zalar P. de Hoog S. Plemenitaš A. (2000). Hypersaline waters in salterns - natural ecological niches for halophilic black yeasts. FEMS Microbiol. Ecol. 32, 235–240. 10.1111/j.1574-6941.2000.tb00716.x 10858582

[B16] Hallsworth J. E. Yakimov M. M. Golyshin P. N. Gillion J. L. D'auria G. De Lima Alves F.. (2007). Limits of life in MgCl_2_-containing environments: chaotropicity defines the window. Environ. Microbiol. 9, 801–813. 10.1111/j.1462-2920.2006.01212.x 17298378

[B17] Hocking A. D. Miscamble B. F. Pitt J. I. (1994). Water relations of *Alternaria alternata, Cladosporium cladosporioides, Cladosporium sphaerospermum, Curvularia lunata* and *Curvularia pallescens*. Mycol. Res. 98, 91–94. 10.1016/s0953-7562(09)80344-47269656

[B18] Hofmeister F. (1888). Zur Lehre von der Wirkung der Salze. Zweite Mittheilung. Archiv. Exp. Pathol. Pharmakol. 247–260.

[B19] Hohmann S. (2009). Control of high osmolarity signalling in the yeast *Saccharomyces cerevisiae*. FEBS Lett. 583, 4025–4029. 10.1016/j.febslet.2009.10.069 19878680

[B20] Jacobson E. S. Ikeda R. (2005). Effect of melanization upon porosity of the cryptococcal cell wall. Med. Mycol. 43, 327–333. 10.1080/13693780412331271081 16110778

[B21] Javor B. J. (1989). Hypersaline environments, in Microbiology and Biogeochemistry, ed Schiewer U. (Berlin: Springer-Verlag Berlin and Heidelberg GmbH & Co. K), 287–287.

[B22] Kocev D. Vens C. Struyf J. Dzeroski S. (2013). Tree ensembles for predicting structured outputs. Pattern Recogn. 46, 817–833 10.1016/j.patcog.2012.09.023

[B23] Kogej T. Gorbushina A. A. Gunde-Cimerman N. (2006). Hypersaline conditions induce changes in cell-wall melanization and colony structure in a halophilic and a xerophilic black yeast species of the genus *Trimmatostroma*. Mycol. Res. 110, 713–724. 10.1016/j.mycres.2006.01.014 16765585

[B24] Kogej T. Ramos J. Plemenitaš A. Gunde-Cimerman N. (2005). The halophilic fungus *Hortaea werneckii* and the halotolerant fungus *Aureobasidium pullulans* maintain low intracellular cation concentrations in hypersaline environments. Appl. Environ. Microbiol. 71, 6600–6605. 10.1128/AEM.71.11.6600-6605.2005 16269687 PMC1287720

[B25] Kogej T. Stein M. Volkmann M. Gorbushina A. A. Galinski E. A. Gunde-Cimerman N. (2007). Osmotic adaptation of the halophilic fungus *Hortaea werneckii*: role of osmolytes and melanization. Microbiology 153, 4261–4273. 10.1099/mic.0.2007/010751-0 18048939

[B26] Kralj Kunčič M. Kogej T. Drobne D. Gunde-Cimerman N. (2010). Morphological response of the halophilic fungal genus *Wallemia* to high salinity. Appl. Environ. Microbiol. 76, 329–337. 10.1128/AEM.02318-09 19897760 PMC2798636

[B27] Kunz W. Henle J. Ninham B. W. (2004). “Zur Lehre von der Wirkung der Salze” (about the science of the effect of salts): Franz Hofmeister's historical papers. Curr. Opin. Colloid Interface Sci. 9, 19–37 10.1016/j.cocis.2004.05.005

[B28] Lenassi M. Gostinèar C. Jackman S. Turk M. Sadowski I. Nislow C.. (2013). Whole genome duplication and enrichment of metal cation transporters revealed by *de novo* genome sequencing of extremely halotolerant black yeast *Hortaea werneckii*. PLoS ONE 8:e71328. 10.1371/journal.pone.0071328 23977017 PMC3744574

[B29] Leong S.-L. L. Lantz H. Pettersson O. V. Frisvad J. C. Thrane U. Heipieper H. J.. (2014). Genome and physiology of the ascomycete filamentous fungus *Xeromyces bisporus*, the most xerophilic organism isolated to date. Environ. Microbiol. [Epub ahead of print]. 10.1111/1462-2920.12596 25142400

[B30] Levatić J. Kocev D. Džeroski S. (2014). The use of the label hierarchy in hierarchical multi-label classification improves performance, in New Frontiers in Mining Complex Patterns, eds Appice A. Ceci M. Loglisci C. Manco G. Masciari E. Ras Z. W. (Heidelberg: Springer International Publishing), 162–177 10.1007/978-3-319-08407-7_11

[B31] Luyten K. Albertyn J. Skibbe W. F. Prior B. A. Ramos J. Thevelein J. M.. (1995). Fps1, a yeast member of the MIP family of channel proteins, is a facilitator for glycerol uptake and efflux and is inactive under osmotic stress. EMBO J. 14, 1360–1371. 7729414 10.1002/j.1460-2075.1995.tb07122.x PMC398221

[B32] McGenity T. J. Oren A. (2012). Life in saline environments, in Life at Extremes. Environments, Organisms and Strategies for Survival, ed Bell E. M. (Walling Ford, UK: CABI International), 402–437.

[B33] Mullakhanbhai M. F. Larsen H. (1975). *Halobacterium volcanii* spec. nov., a Dead Sea halobacterium with a moderate salt requirement. Arch. Microbiol. 104, 207–214. 1190944 10.1007/BF00447326

[B34] Oren A. (1983). *Halobacterium sodomense* sp. nov., a Dead-Sea halobacterium with an extremely high magnesium requirement. Int. J. Syst. Bacteriol. 33, 381–386. 1190944

[B35] Oren A. (2002). Diversity of halophilic microorganisms: environments, phylogeny, physiology, and applications. J. Ind. Microbiol. Biotechnol. 28, 56–63. 10.1038/sj/jim/7000176 11938472

[B36] Oren A. (2011). The halophilic world of Lourens Baas Becking, in Halophiles and Hypersaline Environments: Current Research and Future Trends, eds Ventosa A. Oren A. Ma Y. (Heidelberg;Dordrecht;London;New York, NY: Springer-Verlag), 215–232.

[B37] Oren A. (2013). Life in magnesium- and calcium-rich hypersaline environments: salt stress by chaotropic ions, in Polyextremophiles, eds Seckbach J. Oren A. Stan-Lotter H. (Dordrecht: Springer Netherlands), 215–232.

[B38] Oren A. Gunde-Cimerman N. (2012). Fungal life in the Dead Sea, in Biology of Marine Fungi, ed Raghukumar C. (Berlin Heidelberg: Springer-Verlag), 115–132. 10.1007/978-3-642-23342-5_6 22222829

[B39] Oren A. Gurevich P. Gemmell R. T. Teske A. (1995). *Halobaculum gomorrense* gen. nov., sp. nov, a novel extremely halophilic archaeon from the Dead-Sea. Int. J. Syst. Bacteriol. 45, 747–754. 7547294 10.1099/00207713-45-4-747

[B40] Palkova Z. (2004). Multicellular microorganisms: laboratory versus nature. EMBO Rep. 5, 470–476. 10.1038/sj.embor.7400145 15184977 PMC1299056

[B41] Palkova Z. Vachova L. (2006). Life within a community: benefit to yeast long-term survival. FEMS Microbiol. Rev. 30, 806–824. 10.1111/j.1574-6976.2006.00034.x 16911045

[B42] Pitt J. I. Hocking A. D. (1977). Influence of solute and hydrogen-ion concentration on water relations of some xerophilic fungi. J. Gen. Microbiol. 101, 35–40. 19558 10.1099/00221287-101-1-35

[B43] Pitt J. I. Hocking A. D. (2009). Fungi and Food Spoilage. Dordrecht: Springer.

[B44] Samson R. A. Hoekstra E. S. Frisvad J. C. Filtenborg O. (2002). Introduction to Food- and Airborne Fungi. Baarn: Centraalbureau voor Schimmelcultures.

[B45] Selbmann L. de Hoog G. S. Mazzaglia A. Friedmann E. I. Onofri S. (2005). Fungi at the edge of life: cryptoendolithic black fungi from Antarctic desert. Stud. Mycol. 51, 1–32.

[B46] Sonjak S. Frisvad J. C. Gunde-Cimerman N. (2006). Penicillium mycobiota in Arctic subglacial ice. Microb. Ecol. 52, 207–216. 10.1007/s00248-006-9086-0 16897300

[B47] Sonjak S. Gürsu B. Gunde-Cimerman N. (2010). MgCl_2_ tolerant fungi from the bitterns, in 8th International Congress on Extremophiles AZORES 2010 (Ponta Delgada), 109.

[B48] Stevenson A. Burkhardt J. Cockell C. S. Cray J. A. Dijksterhuis J. Fox-Powell M.. (2014). Multiplication of microbes below 0.690 water activity: implications for terrestrial and extraterrestrial life. Environ. Microbiol. [Epub ahead of print]. 10.1111/1462-2920.12598 25142751

[B49] Tresner H. D. Hayes J. A. (1971). Sodium chloride tolerance of terrestrial fungi. Appl. Microbiol. 22, 210–213. 5096381 10.1128/am.22.2.210-213.1971 PMC377415

[B50] van Der Wielen P. W. Bolhuis H. Borin S. Daffonchio D. Corselli C. Giuliano L.. (2005). The enigma of prokaryotic life in deep hypersaline anoxic basins. Science 307, 121–123. 10.1126/science.1103569 15637281

[B51] Vens C. Struyf J. Schietgat L. Dzeroski S. Blockeel H. (2008). Decision trees for hierarchical multi-label classification. Mach. Learn. 73, 185–214 10.1007/s10994-008-5077-3

[B52] Williams J. P. Hallsworth J. E. (2009). Limits of life in hostile environments: no barriers to biosphere function? Environ. Microbiol. 11, 3292–3308. 10.1111/j.1462-2920.2009.02079.x 19840102 PMC2810447

[B53] Wollenzien U. de Hoog G. S. Krumbein W. E. Urzi C. (1995). On the isolation of microcolonial fungi occurring on and in marble and other calcareous rocks. Sci. Total Environ. 167, 287–294 10.1016/0048-9697(95)04589-S

[B54] Zajc J. Kogej T. Ramos J. Galinski E. A. Gunde-Cimerman N. (2014). The osmoadaptation strategy of the most halophilic fungus *Wallemia ichthyophaga*, growing optimally at salinities above 15% NaCl. Appl. Environ. Microbiol. 80, 247–256. 10.1128/AEM.02702-13 24162565 PMC3911034

[B55] Zajc J. Liu Y. Dai W. Yang Z. Hu J. Gostinèar C.. (2013). Genome and transcriptome sequencing of the halophilic fungus *Wallemia ichthyophaga*: haloadaptations present and absent. BMC Genomics 14:617. 10.1186/1471-2164-14-617 24034603 PMC3849046

[B56] Zajc J. Zalar P. Plemenitaš A. Gunde-Cimerman N. (2012). The Mycobiota of the Salterns, in Biology of Marine Fungi, ed Raghukumar C. (Berlin Heidelberg: Springer-Verlag), 133–158. 10.1007/978-3-642-23342-5_7 22222830

[B57] Zalar P. de Hoog G. S. Schroers H. J. Crous P. W. Groenewald J. Z. Gunde-Cimerman N. (2007). Phylogeny and ecology of the ubiquitous saprobe *Cladosporium sphaerospermum*, with descriptions of seven new species from hypersaline environments. Stud. Mycol. 58, 157–183. 10.3114/sim.2007.58.06 18490999 PMC2104741

[B58] Zalar P. de Hoog G. S. Schroers H. J. Frank J. M. Gunde-Cimerman N. (2005). Taxonomy and phylogeny of the xerophilic genus *Wallemia* (Wallemiomycetes and Wallemiales, cl. et ord. nov.). Antonie van Leeuwenhoek 87, 311–328. 10.1007/s10482-004-6783-x 15928984

[B59] Zhang Y. Cremer P. S. (2006). Interactions between macromolecules and ions: the Hofmeister series. Curr. Opin. Chem. Biol. 10, 658–663. 10.1016/j.cbpa.2006.09.020 17035073

