# Supplementary figures and images for: Chaophilic or chaotolerant fungi: a new category of extremophiles?
Source: Front Microbiol. 2014 Dec 23;5:708. doi: 10.3389/fmicb.2014.00708 (PMC4274975; doi:10.3389/fmicb.2014.00708)

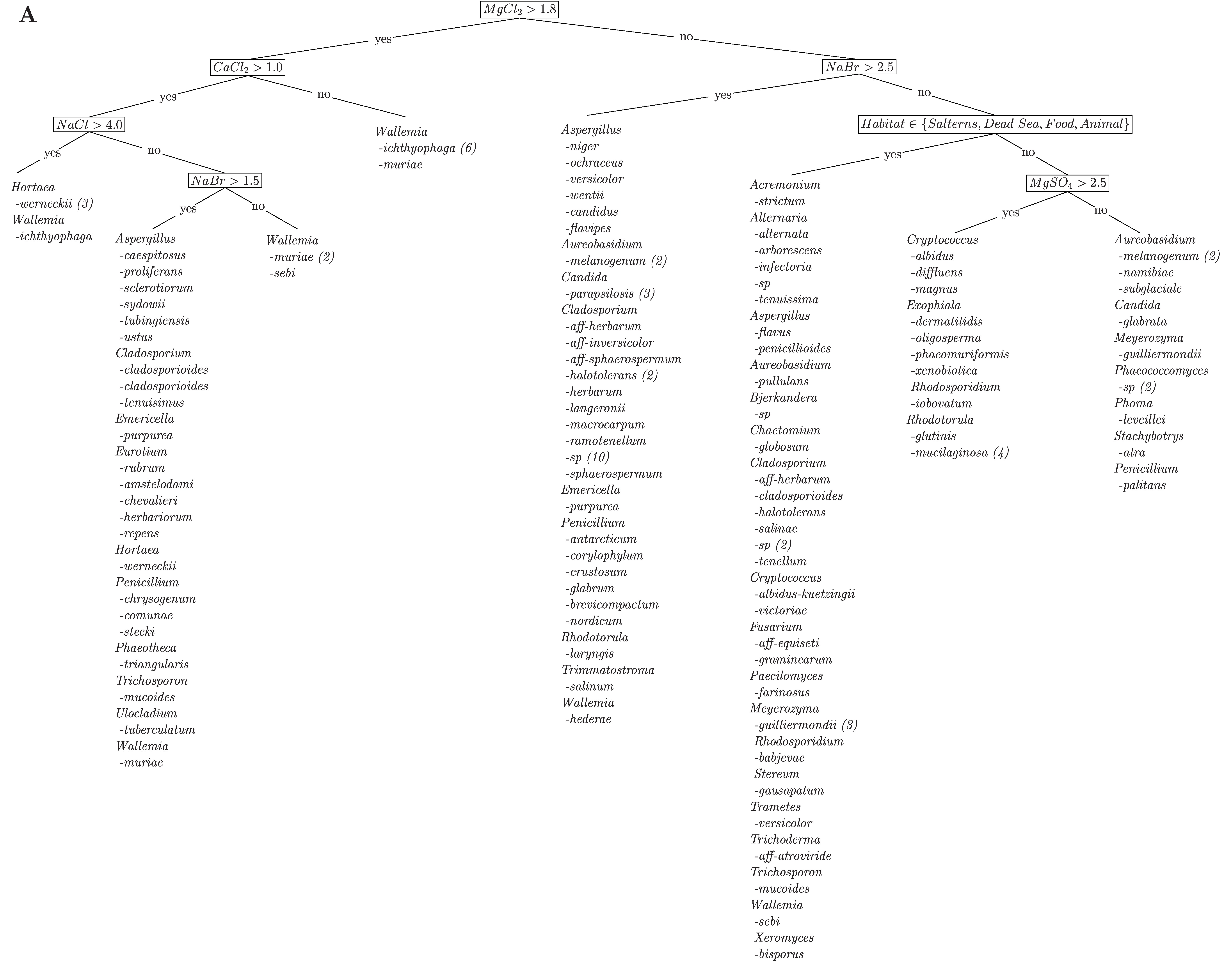

Supplement: Figure S1 — Visualization of the decision trees of fungal species obtained by machine learning tool CLUS. The trees use (A) the habitat (salterns, the Dead Sea, food, freshwater, ice, human, or animal) and the highest concentrations of various salts (NaCl, KCl, MgCl2, CaCl2 NaBr and MgSO4); (B) the habitat, the lowest aw (type of salt), the lowest aw (value) and the highest concentrations of various salts; (C) the habitat, the lowest aw (type of salt) and the lowest aw (value). The target variable was the fungal species (leaves of the decision trees). The numbers in the brackets give the numbers of strains (when more than 1) of each taxon encountered under the given conditions. [file FigureS1-A.TIF]

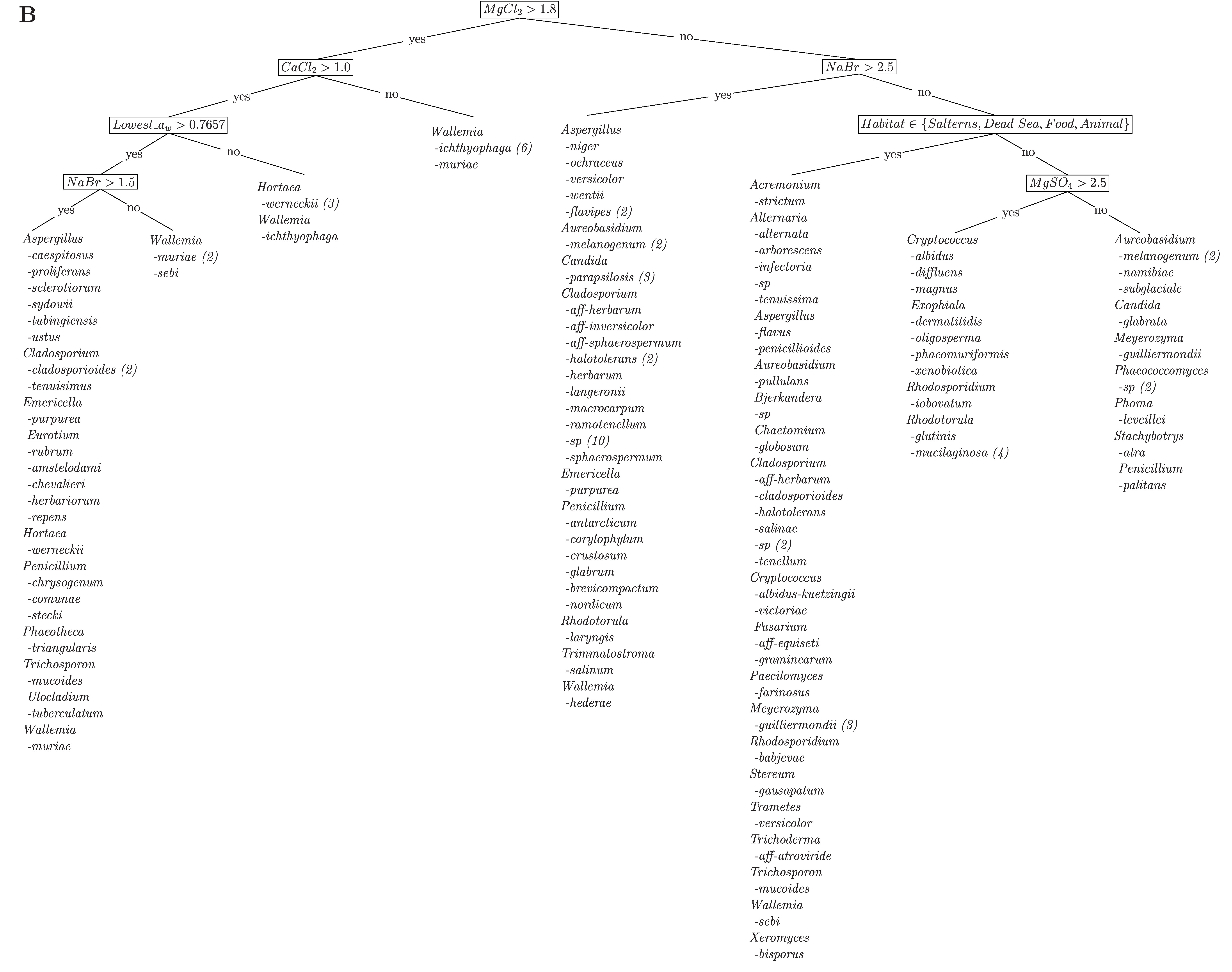

Supplement: Supplementary file 2 [file FigureS1-B.TIF]

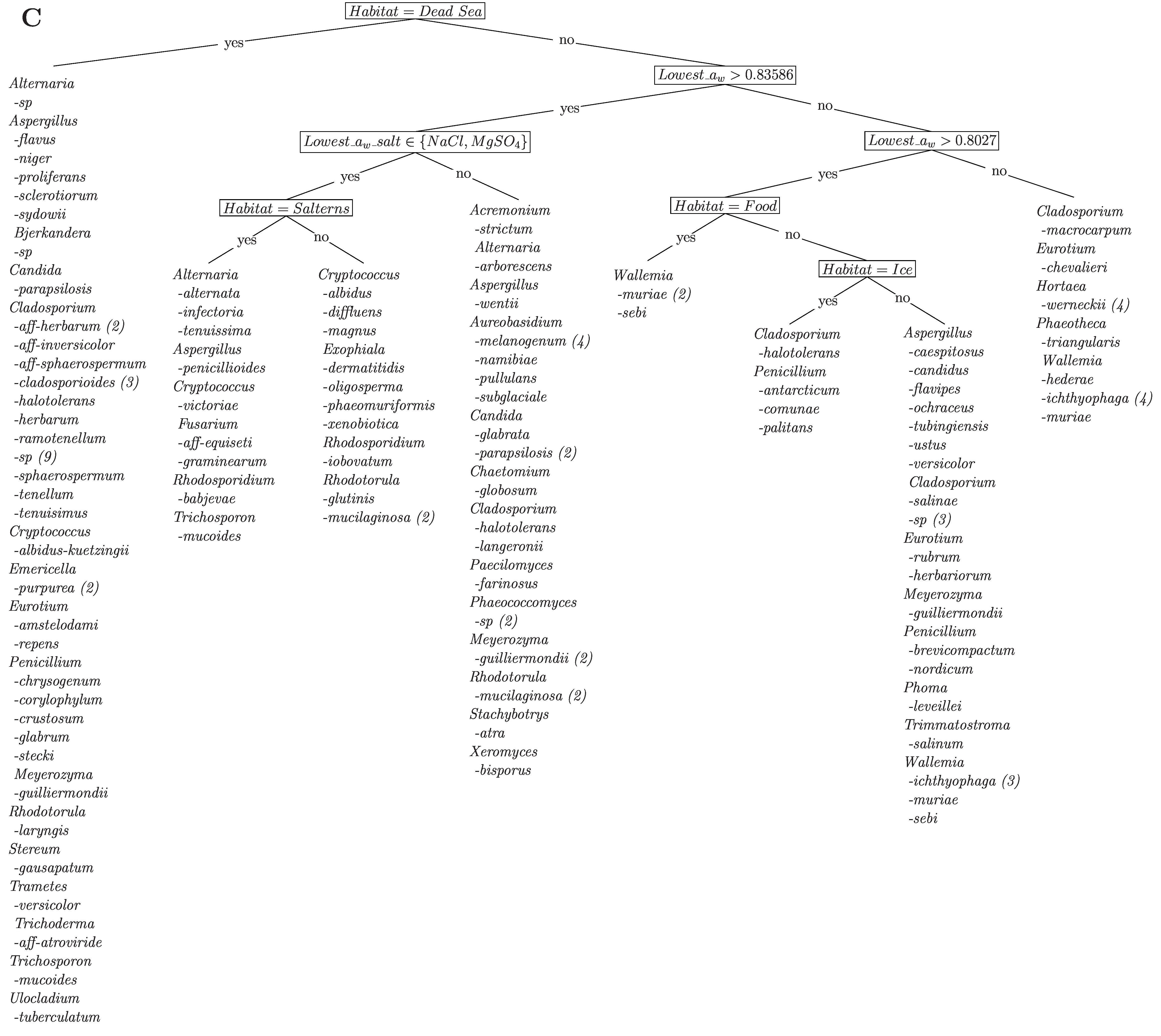

Supplement: Supplementary file 3 [file FigureS1-C.TIF]
